# Use of *Pleurotus eous* Strain P-31 Spent Mushroom Compost (SMC) as Soil Conditioner on the Growth and Yield Performance of *Capsicum annuum* L. and *Solanum lycopersicon* L. Seedlings under Greenhouse Conditions in Ghana

**DOI:** 10.21315/tlsr2018.29.1.12

**Published:** 2018-03-02

**Authors:** M Wiafe-Kwagyan, G T Odamtten

**Affiliations:** Department of Plant and Environmental Biology, University of Ghana, P. O. Box LG 55, Legon, Accra, Ghana

**Keywords:** Sandy Loam Soil, Growth and Yield Performance, Pepper and Tomato Seedlings, Spent Mushroom Compost

## Abstract

The objective of this study was to investigate the influence of spent mushroom compost of *Pleurotus eous* strain P-31 on the growth and yield performance of pepper and tomato seedlings under greenhouse conditions. Sandy loam soil was combined with different percentages of SMC to obtain the following combinations (0, 5, 10, 15, 20, 25 and 30) %. Lower concentrations SMC_5_, SMC_10_ and SMC_15_ promoted vegetative growth (plant height, leaf area, chlorophyll content, number of leaves and axillary branches) of the two test plants. Tomato seedlings grown in SMC_10_ recorded the highest plant height (50.3 ± 7.2cm); leaf area (378.8 ± 1.2cm^2^); number of floral buds (51) and flowers (28) whereas SMC_5_ recorded the highest chlorophyll content 34.1 ± 0.9CCI though SMC_15_ recorded the highest number of leaves (8). Tomato seedlings grown in SMC_30_ produced both the maximum number of fruits (8) with corresponding high weight (34.2 ± 7.7g). Pepper seedlings grown in lower concentrations (SMC_5–15_) recorded the highest plant heights (29.8–30.8cm), chlorophyll content (20.3CCI) and leaf area (53.5–66.2 cm^2^). Although the different combinations of sandy loam soil and SMC did not significantly (*p* ≥ 0.05) affect the number of axillary branches developed; different combinations significantly (*p* ≤ 0.05) affected the number of floral bud, flower and fruit, weight of fruits formed and value of each of these increased with increasing percentage of SMC. Pepper seedlings grown on SMC_30_ recorded the maximum number of floral buds (32.0 ± 3.6), number of flowers (19.4 ± 1.3), number of fruits (10.8 ± 1.2) and weight of fruits (31.9 ± 3.4g). Tomato seedlings raised on SMC_100_ (spent mushroom compost only) and soil only did not significantly (*p* ≥ 0.05) differ from each other however, was statistically significant (*p* ≤ 0.05) from amended sandy loam soil by all criteria investigated. The study shows that SMC provide favourable soil conditioners for the cultivation of fruits, vegetables and foliage crops as it improved growth and yield of tomato and pepper seedlings.

## INTRODUCTION

Throughout the world, mushrooms are the most popular, delicious, nutritious and medicinal vegetables considered as the most promising for crop diversification. This is due their flavour, texture and richness in proteins, minerals, vitamins, low levels of calories with an appreciable amount of unsaturated fats. Mushrooms also accumulate secondary metabolites such as phenolic compounds, polypetides, terpenes, and steroids (Karthika & Murugesan 2015; Souza *et al.* 2016; Thatoi & Singdevsachan 2014; Wiafe-Kwagyan 2014). Mushrooms also have lectins, polysaccharides, polysaccharide-peptides, and polysaccharide-protein complexes which are known to have immunomodulatory and anticancer properties (Sun & Liu, 2009). Mushrooms have been recognised as the alternate source of good quality protein. They are capable of producing the highest quality of protein per unit area and time from agro wastes, which are available at more than one million tonnes per annum in Ghana (FAO 2013). The usefulness of mushrooms and the subsequent production is still on the increase especially with the increase campaign on health, nutritional and medicinal benefits of mushrooms which have all contributed to the growing expansion of the mushroom industry annually (Adedokun & Akuma, 2014; Aderemi *et al*. 2014; FAO 2013). Generally, mushrooms are produced on natural materials taken from agriculture, woodlands, animal husbandry, and manufacturing industries such as hay, straw, horse bedding, poultry litter, corn cobs, corn stover, cotton seed meal, coffee residues (grounds, hulls, stalks and leaves), cocoa pod husk/shells, banana fronds, agave waste, soy pulp to mention but few (Adedokun & Akuma, 2014; Aderemi e*t al*. 2014; Stamets 2000; Wiafe-Kwagyan 2014). Production of mushrooms on these agro-wastes is accompanied by the generation of million tons of residue referred to as spent or used mushroom substrates (sometimes called mushroom soil, recycled mushroom compost, or mushroom compost; (SMC) which remain after the mushroom crop has been harvested (Wiafe-Kwagyan 2014). According to many researchers e.g. (Aderemi *et al*. 2014; Gonani *et al.* 2011; Philippoussis *et al.* 2004; Lau e*t al.* 2003; Wiafe-Kwagyan 2014) it is estimated that huge production of more than 10–50 million metric tonnes of spent mushroom compost is expected to be generated annually worldwide (Gonani *et al*. 2011; Philippoussis *et al*. 2004; Wiafe-Kwagyan *et al*. 2016). Therefore, it has become the subject of great importance as this is a great environmental challenge in terms of its effective disposal (Gonani *et al*. 2011; Philippoussis *et al.* 2004; Wiafe-Kwagyan 2014; Wiafe-Kwagyan *et al.* 2016). Spent mushroom compost (SMC) is often regarded an agricultural waste product with little inherent value, yet there is still value in the substrate as it is rich in nutrients and organic matter that can provide benefits to other agricultural and non-agricultural sectors. However, after suitable pre-treatment spent mushroom compost can completely or partially substitute the growing media for cultivation of different economically important horticultural crops (Roy *et al.* 2015). Spent mushroom compost is also a good source of carbon, nitrogen and other elements. Nitrogen content varies from 0.4%–13.7% with a C: N ratio of 9 to 15: 1 which enhances the growth of plants (Roy *et al*. 2015). SMC which also contains simpler form of protein-rich component formed through modification of agricultural materials by the fungus after few cycles of cultivation, can be used as very good soil conditioners for the cultivation of fruits, vegetables flower and foliage crops (Roy *et al*. 2015). Provision of growing media is one of the most important requirements for agricultural activities, especially for greenhouse plants. One possible solution to this problem is the use of compost which is the most economical and sustainable form of organic waste management (Gonani *et al*. 2011; Nair *et al*. 2005; Çaycı *et al*. 1998).

Apart from using Spent Mushroom Compost as feeding material for vermicomposting, plant disease management, preparation of organic-mineral fertilizer and bioremediation of contaminated soils (Ahlawat *et al*. 2010) there are other alternatives of using SMC. However, these are without any supporting scientific data especially in Ghana and the paucity of information on its application as well as the optimum benefits of using SMC have not exploited (Quartey & Chylkova 2012; Wiafe-Kwagyan 2014). The unscientific ways of using SMC are also creating several problems which include the accumulation of salts in soils and subsequent harmful effect on some crop plants. To date fertiliser cost and the concern for sustainable soil productivity and ecological stability in relation to chemical fertiliser-use has emerged as an important issue (Shahnawaz *et al.* 2009). There is also renewed interest in the use of organic manures such as farmyard manure, compost and green manure as sources of plant nutrients (Roy *et al*. 2015; Marques *et al*. 2014; Uzun 2004; Çaycı *et al*., 1998; Aulakh 1994; Singh *et al.* 1988). These authors observed that manure application improved the availability of some minerals in the soil especially the transfer of nutrients from range land to the crop plants. Other reports also indicate that these materials influence crop yield and affect chlorophyll colouration due to the quantity of nutrients absorbed by the plant from the soil. They are the primary substrate for replenishment of soil organic matter, which on mineralisation supply essential plant nutrients (Walters *et al*. 1992). Spent substrate from different types of mushrooms vary in its physical, chemical and biological properties and each one has its own specific utility. These usages include the following as feeding material for vermicomposting, plants diseases management, preparation of organic-mineral fertiliser and bioremediation of the contaminated soils (Uzun 2004; Aulakh 1994). However presently, farmers in different parts of Ghana are not conversant with the use of SMC as manure or soil conditioner for various field crops and this is because there is paucity of information and any support of the scientific data of its optimum benefits (Wiafe-Kwagyan 2014). Our present study deals with the aspects of correct dose combinations and application of SMS, the effect on vegetative growth and development of tomato and pepper seedlings under greenhouse conditions in Ghana.

## MATERIALS AND METHODS

### Materials and Experimental Methods

This study was carried out in the greenhouse at the Department of Plant and Environmental Biology, University of Ghana Legon. Potted plastic buckets of dimensions 25 cm and 22 cm diameter were used to pot the test seedlings. Pepper (*Capsicum annuum* L. var. Legon 18) and Tomato (*Solanum lycopersicon* Fr. *Lycopersicon esculentum* Mill. var. Wosowoso) seeds were obtained from the Department of Crop Science, School of Agriculture, University of Ghana.

### Collection of Spent Mushroom and Soil Samples

Soil type used for this study was sited at the Haatso soil series, a well-drained sandy loam soil described as Foric Acrisol according to the FAO/UNESCO (1998, 1997). Soil map of the world, revised legend. The experimental site about 76.0m above sea level, situated on Latitude 0ºS 40ºN and longitude 0º 13ºW in the Coastal Savanna agro-ecological zone of Ghana. The annual rainfall is less than 1000mm.

Spent mushroom compost (SMC) derived from mushroom (*Pleurotus eous* Strain P-31) cultured in polyethylene bags on rice straw after a cropping period of 8 weeks. The bags were obtained from the Mycology Unit of the Food Research Institute of the Council for Scientific and Industrial Research, Ghana. Sandy loam soil used for this present study was collected from the University’s Farm in the Legon Botanical Gardens University of Ghana, Legon. The soil sample was taken to the Department of Soil Science, School of Agriculture for authentication, identification and elemental nutrient analysis.

### Experimental Design

Pepper (*Capsicum annuum* L. var. Legon 18) and Tomato (*Solanum lycopersicon* Mil. var. Wosowoso) were used as test plants because they are among the plants most preferred for cultivation in backyard gardens and commercial farms in Ghana. To study the effect of the spent mushroom compost (SMC) on vegetative growth of the two crops, a modified method of (Önal & Topcuoglu, n.d.; Polat *et al*. (2009) and Gonani *et al*. 2011; Çaycı *et al*. 1998) was used. The SMC was first sun dried for 5 days in order to reduce its moisture content and eliminate undesirable resident bio-deterioration agents such as maggot, insects etc. Subsequently the dried SMC was added to loamy soil (Haatso series) collected from university farm in order to obtain the different combinations of soil and SMC in following percentages; SMC_0_ (100% soil; control growing medium); SMC_5_ (5% of SMC to 95% soil); SMC_10_ (10% of SMC to 90% soil); SMC_15_ (15% of SMC to 85% soil); SMC_20_ (20% of SMC to 80% soil); SMC_25_ (25% of SMC to 75% soil) and SMC_30_ (30% of SMC to 70% soil).

SMC in the following ratios SMC_5_, SMC_10_, SMC_15_, SMC_20_, SMC_30_, SMC_100_ were added to sandy loam soil to obtain a total weight (w/w) 6.5 kg for each plastic bucket. Thoroughly mixed of SMC and sandy loam soil the growth medium in each plastic bucket was irrigated with 1 l running tap water. Three weeks old seedlings of either pepper or tomato from a wooden tray were transplanted into each plastic bucket. (5 seedlings per bucket). The potted plants in the plastic buckets were arranged in a randomised complete-block design (RCBD) with five replicates for each of the treatments afore-mentioned above. Five replicates plastic potting buckets of the same size and dimension each was filled with either 6.5 kg SMC, or soil medium served as controls for comparison purposes. Soil: SMC combinations/mixtures served as treated medium. Seedlings were watered every other day with equal volumes of 500 ml of tap water. The following parameters were recorded on weekly intervals: plant height, number of leaves, floral buds, flowers, fruit, axillary branches, leaf area, and chlorophyll content of leaves, stem girth and no. of fruits and weight of fruits.

### Total Chlorophyll Content of Leaves

This was determined after 8 weeks of transplanting seedlings using a chlorophyll meter (Optic Sciences CCM-200 plus Model USA) by attaching the chlorophyll meter knob to five randomly selected leaves of equal age and size and average chlorophyll recorded as Chlorophyll Content Index (CCI).

### Number of Leaves and Total Leaf Area

The number of leaves formed was counted manually per week during the 12 weeks growing period. Leaf area in mm^2^ was determined using a digital leaf area meter (LI-3000C) via attaching five randomly selected leaves of same age and size.

### Plant Height

This was estimated by using a meter rule placed firmly on the surface of substrate to determine the height of the plant in centimeters.

### Determination of Moisture Content, pH of Soil and SMC

Moisture content of loamy soil and spent mushroom compost served as controls was determined using the conventional gravimetric method. 1 g of the sample was weighed in a can using a Digital Computer Scale (ACB plus Adams, Equipment Company Ltd. Milton Keynes, UK). Drying was done in a hot oven (Gallenkamp oven, 300 plus series, England) at 105°C for 3 h allowed to cool in a desiccator and was re-weighed. The cooled sample was returned to the oven for further drying and cooling until no further reduction in weight was obtained. The moisture was expressed as a percentage. The pH of the sandy loam soil and spent mushroom compost served as controls using a pHM92 pH meter (MeterLab^TM^, Radiometer Analytical A/S Copenhagen, Denmark).

### Dry Matter

After 12 weeks of vegetative growth in the potting buckets, the plants were carefully removed from the compost to avoid breakage and were thoroughly washed in running tap water followed by three changes of clean water in order to recover all the roots. The plants (pepper and tomato) were dismembered into shoot and roots, placed in brown paper envelopes for each species before weighing each separately and placing them in an oven (Gallenkamp oven, 300 plus series, England) at 100°C for 24 h and then re-weighed after cooling.

### Mycoflora Profile of Soil and SMC

This was determined using the decimal serial dilution technique up to 1:10^4^ dilutions. One millitre aliquots of the serially diluted 1 g per 100 ml of 0.1% peptone water was mixed with either 20 ml of Cooke’s medium or Dichloran Rose Bengal Chloramphenicol Agar (DRBC) in 9.0 cm sterile petri plates and the plates swirled to obtain uniform distribution of spores. There were three replicates at each dilution level. The plates were incubated at 28 ± 2°C for 7 days after the total number of colonies were calculated in log_10_ CFU/g sample. The species of fungi appearing were identified according to their colour, morphological cultural characteristics as outlined by Barnett and Barry (1972); Samson and Reenen-Hoekstra (1988); Von-Arx (1970). The percentage of individual fungal species resident in the soil and SMC was calculated and represented graphically.

### Proximate Analyses of the SMC

Percentage crude protein, hemicellulose, cellulose and lignin were determined by the conventional Kjeldahl’s method (AOAC 2005) and James (1995). The nutrient detergent fiber and acid detergent fiber were estimated using the method of Van Soest *et al*. (1991).

### Determination of Mineral Element Content

The following elements (N, Ca, Mg, K, P, Cu, Zn, Mn, Pb, Na and Fe) were estimated in the soil and SMC using the conventional methods (Atomic absorption spectrometry, Flame Atomic Emission Spectrometry and Kjeldahl). 250 mg of powdered air dried sample was weighed into a beaker was then placed in ignition muffle furnace (Vectar-furnace, PS3-Sweden) for drying at 400°C for 24 h. Five millitres of hydrochloric acid (HCl) was added to the sample and the solution was dried again; subsequently 5 ml of nitric acid (HNO3) was added. After evaporation, the sample was diluted to 50 ml with water. Sodium (Na) and calcium (Ca) content of the ashed sample were determined by flame photometer. K, P, Mg, Cu, Zn, Mn, Fe, and Pb were determined by Unicam 929 Atomic Absorption Spectrophotometer (AAS) (Model PinAAcle 900T). Nitrogen was estimated using the micro Kjeldahl method (Cunniff 1995).

### Statistical Data Analysis

Data obtained were subjected to statistical analyses using the Statistical Package for Social Sciences (SPSS version 20 for Windows). Significant differences between vegetative parameters measured among plants in treated media (soil: SMC) and controls media were determined using Duncan’s Multiple Range Test (DMRT) at 5% probability.

## RESULTS

### Vegetative Growth

#### Plant height

**Figure 1 (A–D):**
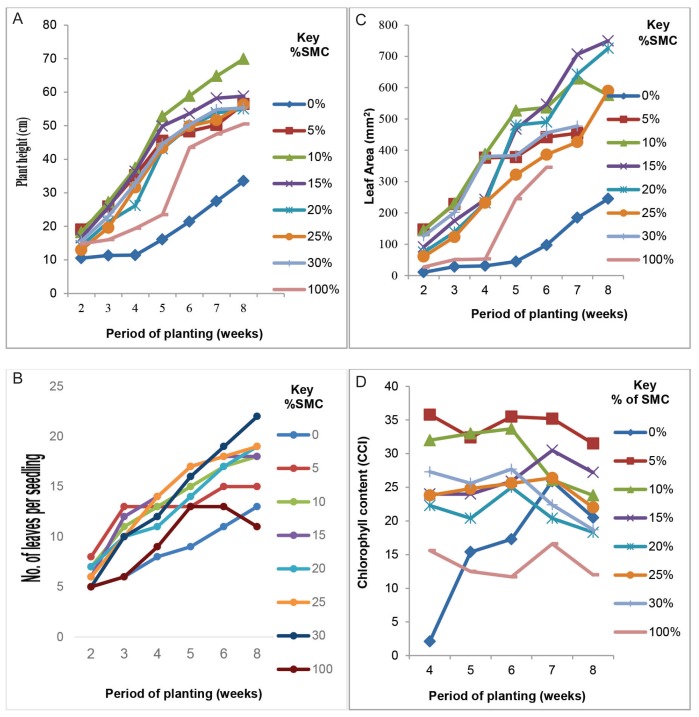
Influence of varying percentages of SMC in soil on the growth and development of tomato seedlings using varying proportions of soil: SMC under greenhouse conditions at 30 ± 2°C for 8 weeks

**Plate 1:**
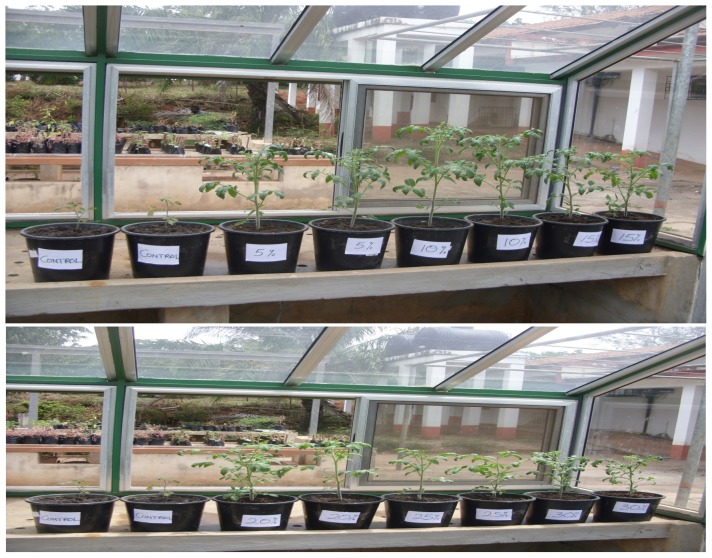
Vegetative growth of tomato seedlings in the indicated Soil: SMC mixtures growing in greenhouse at 30 ± 2°C for 4 weeks after transplanting. (Mg. × 1/10). **Key:** Control = Soil only; 5% = SMC_5_; 10% = SMC_10_; 15% = SMC_15_; 20% = SMC_20_; 25% = SMC_25_; 30% = SMC_30_

Height of tomato seedlings grown in loamy soil amended with SMC at all the different concentrations of SMC (SMC_5–30_) were significantly (*p* < 0.03) higher when compared to that of the control (i.e. those grown in loamy soil only) (Fig. 1A); the height growth of the seedlings planted in SMC_10_ was the highest (69.9 cm) (Fig. 1A & Plate 1).

**Figure 2 (A–D):**
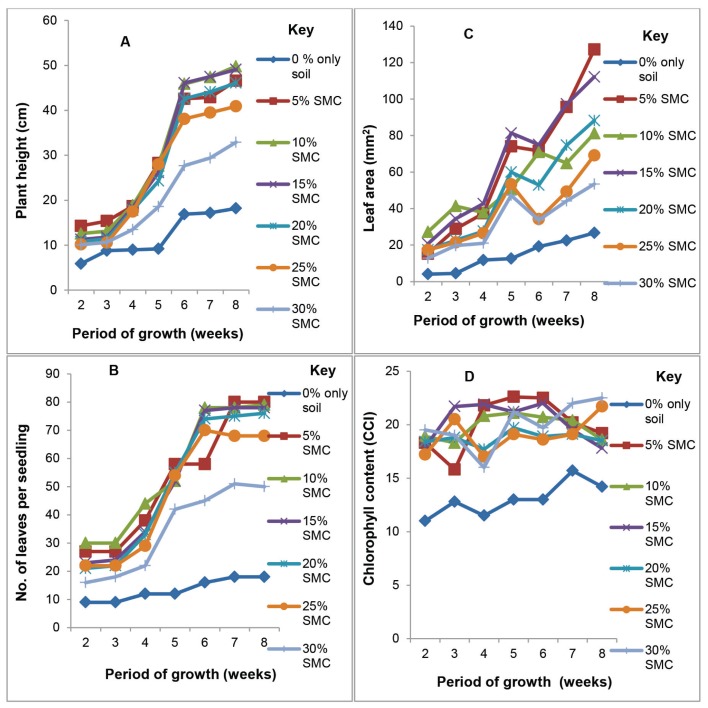
Influence of SMC on the growth and development of pepper seedlings grown in varying proportions of soil: SMC mixtures under greenhouse conditions at 28°C

**Plate 2:**
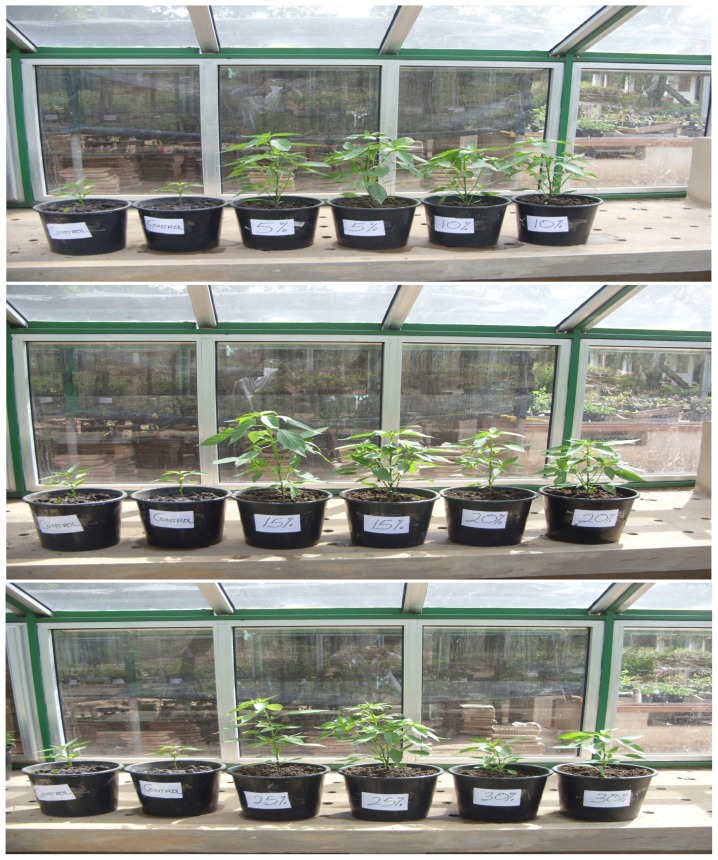
Photographs showing comparative height and leaves formation by pepper plants grown in the indicated mixture of soil and SMC after 6 weeks’ growth at 30 ± 2°C in greenhouse (Mg. × 1/10) **Key:** Control = Soil only; 5% = SMC_5_; 10% = SMC_10_; 15% = SMC_15_; 20% = SMC_20_; 25% = SMC_25_; 30% = SMC_30_

Height of pepper seedlings in pots in all the treatments approximated sigmoid curves (Fig. 2A). There were no statistical differences (*p* > 0.05) in the height of pepper plants grown in sandy loam soil amended with SMC_5–25_ after 8 weeks of growth. Growth of pepper seedlings in unamended loamy soil (control) was the lowest (18.2 cm) and was statistically (*p* < 0.05) different from data obtained in the sandy loam soil medium amended with SMC_5–30_ (Fig. 2A and Plate 2 shows the growth habit of pepper seedlings in pots).

### Number of Leaves and Leaf Area

Number of leaves and leaf area of tomato seedlings grown in loamy soil medium amended with various concentrations of spent mushroom compost (i.e. SMC_5–30_) were also significantly (*p* ≤ 0.05) larger when compared to the values of those grown in the control planting medium (Fig. 1B). Sandy loam soil was amended with SMC at different concentrations (i.e. SMC_5,_ SMC_10_, SMC_15_ etc.) also had significantly (*p* ≤ 0.05) higher effect on the growth/expansion of leaf area in the tomato seedlings (Fig. 1C) whence the leaf area in the seedlings grown in SMC_10_, SMC_15_ and SMC_20_ were the largest with SMC_25_ having the least influence on leaf area expansion; although its effect was significantly lower than the mean leaf area of tomato seedlings grown in only loamy soil planting medium (Fig. 1C).

Leaf formation by pepper plants grown in both control medium (soil only) and loamy soil amended with SMC_5–30_ also followed a near sigmoid curve similar to tomato seedlings. Loamy soil amended with SMC ranging from SMC_5–25_ proportionately increased the number of leaves up to 70–80 per seedling. However, at SMC_30_ there was a dramatic decline in the number of leaves to about 50 in 8 weeks (Fig. 1D). The difference observed were statistically (*p* ≤ 0.05) significant. The differences in the stimulation of leaf production by SMC_5–20_ were initially indistinguishable after 2–6 weeks and remained so until 8 weeks at least for concentration of SMC_5–20_ (Fig. 1D). Leaf area measurements of developing seedling followed the same trend as the plant height. Plants grown in the control medium attained an average area of about 250 mm^2^ in 8 weeks as compared to seedlings grown in pots with 15 and 20% SMC (Fig. 2C) with a leaf area of 700 – 720 mm^2^ (near 3 times). The differences observed after 8 weeks were statistically significant (*p* ≤ 0.05). Maximum net photosynthetic area of the leaf was obtained in plants grown in 10% SMC and was similar to what was obtained for 15 and 20% SMC. Data on leaf area of pepper seedlings grown in soils amended with 5%–30% SMC is presented in Fig. 2B. Mean leaf area of plants cultivated in soil amended with 5%–25% SMC were initially not significantly different (*p* > 0.05) after 2–4 weeks but was significantly different (*p* ≤ 0.05) after 8 weeks. The largest leaf area was obtained in soil amended with 5% SMC which was statistically (*p* ≤ 0.05) different from the leaf areas of plants grown in soil amended with 15% SMC. As the SMC: soil ratio increased the leaf area declined (Fig. 2B). Interestingly, the smallest leaf area ≤ 20 mm^2^ was recorded in plants growing in the unamended native soil.

### Chlorophyll content of leaves of approximately uniform and chronologically at metabolic ages

Chlorophyll content of tomato plants significantly (*p* ≤ 0.05) decreased with increasing levels of SMC in soil in the following descending order 5% >10% >15% >20% >25% >30%. However, the lowest chlorophyll content recorded in soil amended with SMC was still superior statistically (*p* ≤ 0.05) to that of the control (Fig. 1D). Chlorophyll content of leaves of pepper plants grown in soil amended with 5% – 30% SMC was erratic, albeit these potted plants produced the highest recorded chlorophyll content ranges from 18 – 22 CCI in 8 weeks. Chlorophyll levels in leaves of pepper plants sown in unamended soil (control) was 11–15 CCI and which never approximated those in the amended soils (18–22 CCI) throughout the 8 weeks growth. The difference observed were statistically significant (*p* < 0.05) (Fig. 2C).

### Dry matter

The lowest dry matter of tomato plant was obtained in the unamended soil which recorded shoot weight of 90.3 ± 5.9 g and roots weight of 45.3 ± 2.9 g (Table 2). There was commensurate increase in dry weight of shoot and roots as the concentration (%) of SMC increased from 5%–25% and thereafter declined to 94.9 ± 3.90 g for the shoot and 48.9 ± 2.48 g for the roots in pots containing spent mushroom compost only (100% SMC). Dry matter accumulation by pepper shoots followed similar a trend as that of the tomato plant. Accumulation of dry matter by pepper plants increased with increasing % SMC (5%–30%) potting medium. The highest dry matter of the shoot system of pepper plants recorded (146.6 g) was obtained in 30%SMC whereas the least (27.1 g) was recorded on unamended soil (only loamy soil) (Table 1). Although, pepper plants grown in soils amended with different levels of SMC (5%–30%) increased the dry matter of pepper seedlings, it did not follow a similar trend as observed in the shoot system. In the case of tomato there was positive correlation between dry weights of shoot and root in all the growing media (0 – SMC_100_) (Table 3). On the contrary, increased in dry matter accumulation of pepper seedlings shoot only commensurate with increased in dry weight roots of seedlings grown on (soil only/SMC_0_, SMC_5_ and SMC_10_). Thereafter increased in dry weight of shoot did not correspond with increased dry weight of root (SMC_15–30_) Table 1 as was observed for tomato seedlings. The highest dry matter of root system recorded was 46.9 g (10% SMC) while it decreased to 45.2 g for 15% SMC and further declined to 40.7 g for 30% SMC. The lowest (24.7 g) dry matter of pepper was recorded in soil only (unamended soil) (Table 1). Dry matter accumulation obtained by both tomato and pepper plants grown in soil amended with different levels of SMC were statistically higher (*p* ≤ 0.05) than that obtained in loamy soil only (Tables 1 & 2).

### Mycoflora profile of soil and SMC

**Figure 3:**
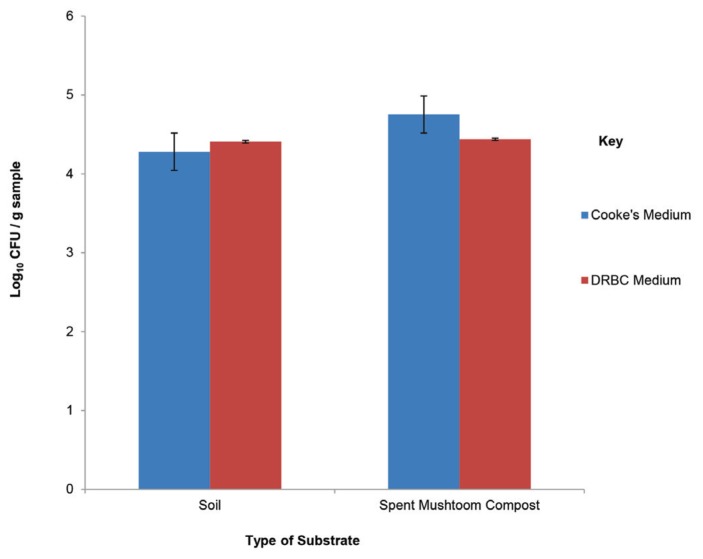
Initial mycoflora population resident in raw soil and raw SMC used for the cultivation of cowpea, pepper and tomato.

The total fungal population in the soil using two media (Cooke’s medium and DRBC) varied between 4.2–4.3 log_10_ CFU/g sample; in the SMC the fungi population estimated in the two-media varied from 4.3–4.5 log_10_ CFU/g. There was therefore no statistically significance difference (*p* > 0.05) between the mycoflora population recorded on the two media (Fig. 3).

**Figure 4:**
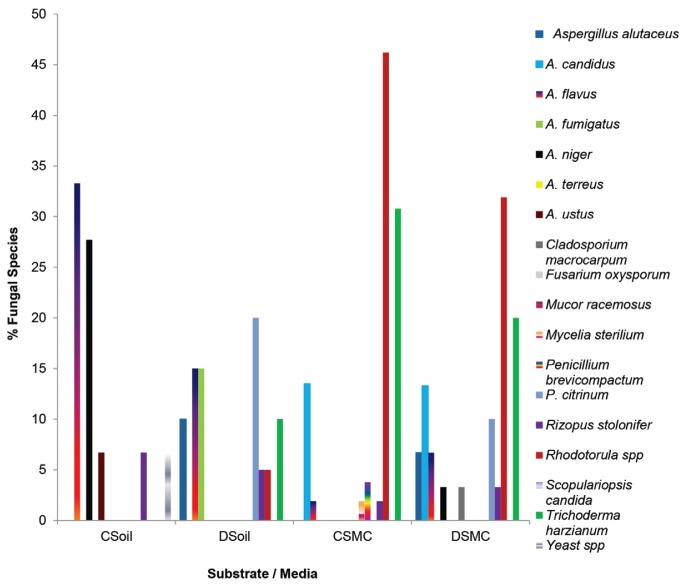
Mycoflora profile of soil and SMC estimated on two mycological media at 28 ± 2°C for 7 days. **Key:** C Soil: Cooke’s Medium (Soil); C SMC: Cooke’s Medium (Spent Compost) **D Soil:** DRBC Medium (Soil); D SMC: DRBC (Spent Compost)

The use of two isolation media enabled a wider spectrum of fungi to be detected. Eighteen (18) different fungi belonging to 9 genera (*Aspergillus, Cladosporium, Fusarium, Mucor, Penicillium, Rhizopus, Rhodotorula, Trichoderma, Scopulariopsis*) other yeasts and *Mycelia sterilia* were encountered in both samples (Fig. 4). *Aspergillus flavus, A. niger, Penicillium citrinum, Rhizopus stolonifer* and *Trichoderma harzianum* were dominant in the soil sample while *Rhodotorula, T. harzianum, P. citrinum, Aspergillus candidus* and *A. alutaceus, A. flavus* were prominent residents (Fig. 4). *A. flavus* constituted 5%–48% of the population while *T. harzianum* contributed 20%–30% of the total mycoflora. On the other hand, *Rhodotorula* provided 33%–47% while the population of *P. citrinum* was low (8%–9%) (Fig. 4).

### Mineral Elements in Loamy Soil and SMC Samples

The soil sample did not contain Cu and Fe, although Zn, Mn, Pb, Ca, Mg, Na, P, K and N (Table 2) were detected in low concentrations. The SMC did not also contain Fe though it contained Zn, Cu, Mn, Pb, Ca, Mg, Na, P, K and N all of which were detected in low concentrations. The pH of the soil and SMC were pH 6.8 and 6.6 respectively and therefore well within the favourable range for growth of tomato and pepper.

## DISCUSSION

SMC is currently disposed of as waste and constitutes an environmental waste problem in Ghana. However, there can be economic benefit from its use in agriculture as bio-fertiliser, if properly harnessed. Maher *et al*. (2000) showed that SMC increased soil organic matter content and improved soil structure. It is also effective source of K and P and provides trace elements for plant growth and as well as contribute to nitrogen nutrition. Other researchers have also shown that SMC can be used with beneficial effect in field crop production. For example; Peksen and Uzun (2008) and Crecchio *et al*. (2001) have shown that SMC is rich in organic matter and constitutes an important source of macro and micro nutrients for plants and microorganisms thereby increasing the soil microflora, soil biological activity and enhance soil enzyme activity. SMC also contains calcium carbonate which provides short time buffering of acid waters and elevates soil pH (Gonani *et al*. 2011; Rupert 1994). Earlier studies by Alsadon *et al.* (2006) show that leached SMC could significantly (*p* ≤ 0.05) increase plant height and number of fruits produced by cucumber (*Cucumber sativus* c.v. Super dominos at the level of sandy-loam soil with 15% of 20% SMC. Peksen and Uzun (2008) investigated the effect of chemical composition of seedling media prepared by SMC on seedling growth and development of kale (*Brassica oleraceae* L. var. acephale D.C. cv. Temel) and broccoli (*Brassica oleracea* L. var. italica L. cv. Greenpeace F1). Their results revealed that SMC and peat or SMC could be used as seedling media for both kale and broccoli. Kadiri and Mustapha (2010) reported the effect of SMC from the cultivation of *Lentinula subnudus* as soil conditioner for cowpea and tomato. Their findings revealed that composted spent mushroom substrate mixed with loamy soil produced greater vegetative growth and yields of both vegetables than loamy soil (controls). Studies by Ogbonna *et al*. (2012) demonstrated that SMC could be used to improve growth and yield of maize (*Zea mays*) in Nigeria.

Data from the present studies show that the SMC of *P. eous* contained the following: 90.8 ± 1.79% dry matter, 7.76 ± 0.48% crude protein, 32.34 ± 1.97% cellulose, 7.26 ± 1.94% lignin, 53.3 ± 6.0% NDF and 50.42 ± 4.64% ADF (Wiafe-Kwagyan 2014), calcium, potassium, nitrogen, sodium (Table 1) and some heavy metals such as copper, iron, manganese, lead and zinc (Table 1) which could influence the growth of the two test plants either positively or negatively.

These results confirm that SMC at 10% mixture with soil was the best in stimulating plant height of tomatoes. The height of plant growing in 5%–25% SMC were similar and did not differ significantly (*p* ≥ 0.05) although it did not approximate the best growth in height in 10% SMC after 8 weeks (Fig. 1A, Plate 1). The poorest growth was obtained in plants grown in soil only. Similar trends were obtained for number of leaves, leaf area, per plant and dry weight of shoot and root (Table 3; Fig. 1B–C). Chlorophyll content in the leaves however, decreased with increasing percentage of SMC mixture with soil. Although beneficial, increased concentration of SMC beyond 20% may be inhibitory and may also limit growth beyond a threshold value.

Pepper plants responded differently in a near sigmoid growth curve. As concentration of SMC increase from 5%–20% there were no statistically significant differences (*p* ≥ 0.05) between the performances of the plants in terms of height, number of leaves and leaf area produced per plant (Fig. 2A–C). However, a concentration of 30% SMC severely depressed height, number of leaves and leaf area and was statistically significant (*p* ≤ 0.05) from pepper seedlings grown on SMC_5–25_. Albeit it performed better than seedlings grown in sandy loam soil only. Growth of pepper plants in soil only medium (control) was lower by all criteria than plants grown in soil media amended with (5–30) % SMC (Fig. 2A–C). Total chlorophyll content in pepper plants recorded in unamended soil (soil only) was the lowest (11–16 CCI) as compared to 18–22 CCI obtained for soil amended with SMC (5–30) %. Changes in total chlorophyll content of plant grown in potting media containing SMC (5–30) % was erratic over the 8 weeks period. Photosynthesis occur in two phases, a light and dark reactions in what is termed photosystem I and II within the chloroplasts. Reaction sites for light and dark reactions could have been influenced by the levels of mineral elements and nutrients and chlorophylls ‘*a* and *b*’. It is conjectured that; total growth of the plant could be affected by the efficiency of the coordinated reactions in photosystems I and II. Although only total chlorophyll was determined in this study, future studies could follow up changes in chlorophylls ‘a and b’ content of the plants in the variously formulated substrate for growth of both tomato and pepper which may provide information to elucidate the changing fortunes of chlorophyll as concentration of SMC increases. Guo and Chorover (2004) stated that high concentrations of elements may limit or inhibit growth beyond a threshold concentration.

Recent studies shows the influence of SMC from oyster and button mushroom on growth yield of *Capsicum annuum* L. and *Solanum tuberosum* L. by Roy *et al*. (2015); Altindal and Altindal (2015) recorded growth promotion in terms of height of plant, number of branches, yield and overall growth. It was conjectured that SMC had a role in the mobilisation of soil phosphate which was evident by decrease in soil phosphate level and increase in root and leaf phosphate (Roy *et al*. 2015; Altindal & Altindal 2015).

Marques *et al*. (2014) used 42%–48% spent mushroom compost substrate for the cultivation of lettuce seedling (*Lactuca sativa* L) and showed that it provided the most adequate conditions for the growth and development of crisp head lettuce seedlings. Idowu and Kadiri (2013) used the SMC of *P. ostreatus* to cultivate okra (*Abelmoscus esculentus* Moench) and found that the growth and yield of okra (fresh and dry weight of fruits and number of okra fingers) was significantly enhanced. Data from this study suggest that spent mushroom compost of *P. eous* used as a soil conditioner to a certain level can be used as a bio-fertiliser for the enhancement of growth and yield of tomato and pepper at least under greenhouse conditions in Ghana.

Furthermore results of this study also show that, SMC of *P. eous* contained high dry matter (90.8 ± 1.79%), protein (7.76 ± 0.48%), cellulose (32.34 ± 1.97%), lignin (7.2 ± 1.94%), NDF (53.3 ± 6.07%), and ADF (50.42 ± 4.64%) rich enough for utilisation by the resident fungi for lignolytic, cellulolytic, pectinolytic, and proteolytic metabolic activities “*in situ”* (Figs. 1 and 2) thereby releasing nutrients to augment the presence of mineral such as K, Na, Ca, N, P, Fe, Mg, Mn, P and Zn in the soil: compost mixtures to support growth of the tomato and pepper seedlings (Table 3). In addition, SMC contains calcium carbonate which provides short-term buffering of acid waters which elevates soil pH (Gonani *et al*. 2011; Wiafe-Kwagyan 2014).

## CONCLUSION

Current findings indicate that both tomato and pepper seedlings raised on different concentrations of SMC (5, 10, 15, 20, 25 and 30) % amended with sandy loam soil supported vegetative growth (plant height, number of leaves, leaf area, chlorophyll content) and fruit yield. SMC_10_: sandy loam soil medium (0.6 kg SMC: 5.85 kg sandy loam soil combination) was the best suitable medium to support optimum vegetative growth of both test plants. These nutrients acted in concert to promote growth of the two plants in the greenhouse at least to a certain level of concentration of soil with SMC. The pH of the soil and spent mushroom compost were within the pH ranged of 6.6 and 6.8 which are well pH’s suited for growth of both tomato and pepper. Therefore, application and use of SMC in cultivation of these vegetables for field trial is highly recommended.

## Figures and Tables

**Table 1:** Dry matter accumulation by pepper seedlings after 12 weeks of growth in pots containing different indicated percentage of Soil: SMC mixtures in the greenhouse at 28°C–32°C

Substrate treatment (%SMC)	Mean dry weight (g) ±SE	

	Shoot system	Root system
0^*^	27.1 ± 1.25^a^	24.7 ± 2.66^a^
5	70.0 ± 1.65 ^b^	41.5 ± 2.13^b^
10	89.5 ± 5.38^c^	46.9 ± 2.39^b^
15	98.7 ± 3.92^d^	45.2 ± 3.12^b^
20	103.8 ± 2.66^e^	44.4 ± 3.86^b^
25	133.8 ± 16.44^f^	42.4 ± 3.81^b^
30	146.6 ± 13.56^g^	40.7 ± 1.88^b^

Key 0^*^: Unamended Loamy Soil (soil only; control)

Figures with same letter in a column are not significant (*p* ≥ 0.05)

**Table 2:** Physicochemical properties and elemental content of soil and spent compost used as growing medium.

Physical and Chemical Properties	Growing Medium	

Mineral Concentration (mg/kg)	Soil	SMC
Zn	0.016	0.017
Cu	0.000	0.003
Mn	0.137	0.013
Pb	0.137	0.013
Ca	0.471	0.597
Mg	0.444	0.773
Fe	0.000	0.000
Na	0.217	0.196
P	0.890	0.795
K	0.118	0.281
N	0.819	0.978
% Dry Matter	0.98	11.51
pH	6.80	6.60

**Table 3:** Dry matter after 12 weeks of growth in pots containing indicated percentage of Soil: SMC mixtures under greenhouse condition at 28°C–32°C

Substrate Treatment (%SMC)	Mean dry weight (g) ±SE	

	Shoot system	Root system
0^*^	90.3 ± 5.86^a^	45.3 ± 2.99^a^
5	160.6 ± 6.3^b^	83.6 ± 5.13^b^
10	185.5 ± 3.8^c^	92.3 ± 2.20^c^
15	185.2 ± 4.0^c^	84.1 ± 1.37^b^
20	167.9 ± 2.8^d^	82.1 ± 2.52^b^
25	165.4 ± 5.7^d^	78.8 ± 4.10^d^
30	159.0 ± 7.3^e^	77.0 ± 2.79^d^
100	94.9 ± 3.9^a^	48.9 ± 2.48^a^

Key 0^*^: Unamended Loamy Soil (soil only; control)

Figures with the same letter in a column are not significant (*p* ≥ 0.05)
